# Engineering Mosaic T7 Bacteriophages for Dual Peptide Display to Enable Cellular Targeting and Ligand‐Directed Protein Capture

**DOI:** 10.1002/biot.70322

**Published:** 2026-10-28

**Authors:** Luca Marchetti, Kristina Põšnograjeva, Prakash Lingasamy, Karlis Pleiko, Ivar Ilves, Tambet Teesalu

**Affiliations:** ^1^ Laboratory of Precision and Nanomedicine Institute of Biomedicine and Translational Medicine University of Tartu Tartu Estonia; ^2^ National Institute of Research and Innovation Riga Latvia; ^3^ Institute of Technology University of Tartu Tartu Estonia; ^4^ Materials Research Laboratory University of California Santa Barbara California USA

**Keywords:** bacteriophage engineering, homing peptide, molecular targeting, multifunctional nanoparticles, protein pull‐down, receptor identification, T7 phage display

## Abstract

Integrating molecular recognition, tracing, and interactome capture within a single nanoparticle could allow for probing, manipulation, and control of complex biological systems. Achieving such multifunctionality within a single well‐defined nanostructure remains challenging. T7 bacteriophage‐based biological nanoparticles provide a robust and genetically programmable scaffold; however, conventional T7 display systems typically accommodate only one exogenous peptide species per phage particle, limiting multifunctional and combinatorial applications. Here, we describe a mosaic phage platform that enables the simultaneous display of two peptides on separate T7 coat protein subunits. By combining genome‐encoded and plasmid‐driven expression of peptide‐fused capsid proteins, the system provides a modular route for assembling multifunctional phage nanoparticles. We generated mosaic T7 phages co‐presenting the neuropilin‐1 (NRP‐1)‐binding peptide RPARPAR alongside either a HA‐tag or a Twin‐Strep (Tstrep) tag affinity handle. These engineered phages specifically target NRP‐1‐expressing cells and enable immunofluorescence visualization and pull‐down of NRP‐1 from complex lysates. Furthermore, a random peptide T7 phage library co‐displaying the constant lung‐homing peptide RPARPAR shows preferential pulmonary accumulation in vivo, demonstrating utility for combinatorial targeting and tissue‐directed phage selection. Collectively, this dual‐display approach expands phage display by enabling cell targeting, visualization, molecular interactome probing, and in vivo tissue homing within a single genetically encoded nanoparticle platform.

## Graphical Abstract and Lay Summary

A mosaic T7 phage platform enables simultaneous display of two independent peptides on the same phage particle. The modular design integrates receptor targeting, protein capture, and tissue‐directed in vivo phage display, establishing a versatile methodology for multifunctional phage engineering, receptor identification, and next‐generation combinatorial library design.

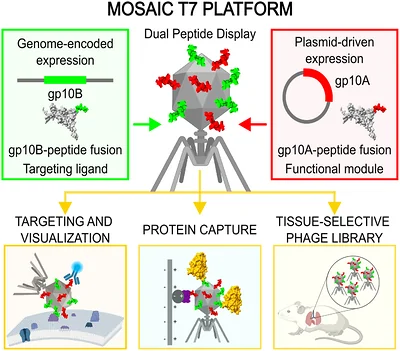

## 1 Introduction

Complex biological functions rely on intricately orchestrated molecular interaction cascades. In nanobiotechnology, co‐displaying distinct peptides or other interaction moieties can generate synergistic effects. Dual‐functionalized synthetic nanoplatforms have been used to couple receptor binding to drug delivery [1, 2], to integrate tumor‐homing peptides with imaging probes for noninvasive diagnostics, or to combine targeting domains with immune‐activating epitopes to enhance antitumor responses [3, 4]. Beyond proof‐of‐concept demonstrations, multifunctional nanoplatforms hold considerable translational potential. By integrating recognition, signaling, and therapeutic functions into a single construct, they can improve selectivity, minimize off‐target effects, and enhance therapeutic indices. In oncology, for example, dual‐ligand nanoparticles have been engineered to navigate heterogeneous tumor microenvironments, sequentially binding to vascular and stromal targets to improve tissue penetration and payload release [5]. In neuroscience, multifunctional constructs are being explored to cross the blood‐brain barrier and deliver neuroactive agents with regional specificity [6]. From a bioengineering perspective, programmable platforms that present multiple ligands in controlled ratios and orientations provide a versatile toolkit for studying multivalent interactions, receptor clustering, and combinatorial signaling. These systems also serve as testbeds for designing next‐generation biologics and targeted nanocarriers capable of responding dynamically to complex biological milieus.

Bacteriophage‐based nanoplatforms offer a bridge between molecular engineering and in vivo functionality. Bacteriophages can be genetically engineered to display peptides as fusions to the coat proteins in a controlled manner, and iterative screening of combinatorial libraries enables rapid identification of ligands for clinically relevant receptors [7, 8, 9, 10, 11]. Phage biopanning can be performed on targets of varying complexity from purified molecules to cultured cells and even in vivo systems yielding homing peptides that are directly relevant for affinity targeting of nanoparticles (NPs), as phages themselves are biological NPs. Among display systems, filamentous bacteriophages such as M13 have been widely used due to their ease of genetic manipulation and extensive use in antibody library construction [12, 13]. Although M13 supports single, dual‐, and even triple‐peptide display, its high aspect ratio limits their ability to emulate the in vivo behavior of clinically relevant nanoparticles, which are typically spherical or only mildly elongated. Recently, M13 was engineered to co‐display two functional peptides, a sensory neuron‐homing motif and a neurotrophic mimic, resulting in selective promotion of sensory innervation in vivo [14]. Plant virus‐derived nanoparticles and virus‐like particles (VLPs) have similarly emerged as highly programmable nanoscale scaffolds, with recent engineering strategies enabling multivalent and mosaic presentation of distinct functional moieties [15, 16]. These platforms have demonstrated broad potential for targeting, imaging, drug delivery, and other biomedical applications [17]. Bacteriophages share many of these attributes while offering a distinctive advantage for ligand discovery, as the physical linkage between displayed peptides and their encoding genetic information enables recovery, amplification, and iterative selection of functional binders from complex libraries.

Among bacteriophage platforms, the icosahedral T7 phage, with its ∼60 nm nucleocapsid [18, 19], more closely matches the dimensions of synthetic therapeutic nanoparticles, making it a superior platform for studying how ligand functionalization influences tissue and subcellular biodistribution as well as interactome profiles. As a result, T7 has become the dominant platform for identifying tissue‐ and disease‐specific homing peptides and for mapping vascular ZIP codes [20, 21]. T7 phages also resemble small VLPs such as MS2 or Qβ (∼27–30 nm), which have been widely deployed for vaccine display, RNA delivery, and imaging applications [22, 23, 24, 25]. T7 particles assemble in the cytoplasm and are released in bulk upon host cell lysis, enabling display of a broader diversity of peptides than filamentous phages and allowing robust, scalable amplification [26, 27]. The double‐stranded DNA genome provides genetic stability while tolerating relatively large foreign inserts, a critical property for accommodating complex peptides or protein domains [28, 29]. Structurally, the T7 capsid is composed of the major coat protein gp10A and its extended form gp10B, the latter generated by a translational frameshift [30, 31]. In the T7Select Phage Display System (Novagen), the phage genome is engineered to incorporate foreign peptide‐encoding DNA sequences that direct expression of the inserted peptides as C‐terminal fusions to gp10B [32, 33, 34]. Current T7 phage display systems are generally limited to the multivalent presentation of a single peptide sequence per phage particle. While this strategy has been highly effective for ligand discovery, expanding display formats to allow the co‐presentation of multiple ligands provides additional versatility, including multiplexed receptor targeting, multimodal detection, and integration of functional modules for both in vitro and in vivo applications.

Here, we report development of a dual‐display mosaic phage platform for bacteriophage T7 to co‐present distinct peptides on the same phage particle. Our strategy combines genome‐encoded fusion of one ligand to gp10B with plasmid‐driven expression of a second ligand fused to gp10A. During phage assembly, both coat protein variants are incorporated into the same phage, generating mosaic phage particles. We show that dual‐display mosaic phage preserves receptor‐specific binding while enabling simultaneous molecular tracking and affinity‐based protein isolation, and that it can be seamlessly integrated with downstream proteomic and cell‐surface interactomics workflows. This modular architecture extends the capabilities of conventional T7 phage display beyond single‐ligand formats, providing a versatile platform for ligand discovery, receptor mapping, generation of tissue‐selective phage libraries, and future translational applications in molecular medicine.

## 2 Results

### 2.1 Engineering Genetic Modules for Dual‐Display Mosaic T7 Phage

To implement the dual‐display mosaic phage design, we first constructed the genetic components required for simultaneous presentation of two distinct ligands on the T7 capsid. As the source of the gp10A coat protein, we isolated the p5403 plasmid from *E. coli* BLT5403 cells; this plasmid carries the wild‐type gp10A gene under a T7 promoter and provides a supply of gp10A in trans during phage assembly. To facilitate cloning of exogenous peptide‐coding sequences at the C‐terminus of gp10A, we engineered a modified version of the plasmid, p5403‐MCS, by introducing a multiple cloning site (MCS) at the 3′ end of the gp10A open reading frame. This design created a platform for constructing plasmids driving the expression of either wild‐type gp10A (pMosWT) or gp10A fused to distinct peptide motifs (Figure S1). In addition to pMosWT, we generated four peptide‐fusion variants: pMosRPAR, pMosHA‐tag, pMosTstrep and pMosTetraCys, each encoding gp10A C‐terminally fused to its respective targeting or affinity tag peptide. The RPAR insert corresponds to the CendR‐motif peptide RPARPAR, a well‐characterized ligand that, when displayed at the C‐terminus of a polypeptide chain, binds neuropilin‐1 (NRP‐1) [35]. The HA‐tag (YPYDVPDYA) is an epitope tag derived from the human influenza hemagglutinin protein and widely used for antibody‐based detection, while the Twin‐Strep (Tstrep) tag (WSHPQFEK–GGGS–WSHPQFEK) is a high‐affinity peptide tag enabling reversible binding to Strep‐Tactin XT for purification and pull‐down applications. The TetraCys peptide (CCPGCC) forms a fluorescent complex upon binding the biarsenical fluorophore FlAsH‐EDT_2_ [36], enabling quantitative assessment of peptide incorporation into the capsid.

In parallel, we generated the complementary gp10B module by inserting DNA sequences encoding RPAR, HA‐tag, TetraCys or the heptaglycine cyclic peptide (cG7c) into the T7Select415‐1b phage genome. These inserts direct expression of gp10B‐peptide fusions from the phage genome to allow for display of the gp10B‐fused peptides on assembling phages. Together, the plasmid‐based gp10A variants and the genome‐encoded gp10B fusions provided the genetic framework for generation of mosaic T7 phages co‐displaying two independent peptide ligands.

### 2.2 Mosaic Phages Preserve Receptor Binding In Vitro

Under mosaic dual‐display conditions, different peptides presented on separate coat proteins may sterically hinder or otherwise interfere with one another's activity. To evaluate whether the functionality of a homing peptide is preserved in a mosaic configuration, we generated monospecific WT and RPAR phages in non‐transformed BL21 bacteria, as well as mosaic WT/RPAR T7 phages in which the wild‐type coat protein was encoded by the phage genome and the RPAR‐fused coat protein was supplied in trans from the pMosRPAR plasmid (Figure 1a). SDS‐PAGE analysis showed two distinct bands in the mosaic preparation, corresponding to the lower‐molecular‐weight WT coat protein and the higher‐molecular‐weight RPAR‐modified variant, whereas the WT and RPAR single‐display phages each exhibited a single band at the expected position (Figure 1b). These results confirm successful co‐assembly of WT and RPAR‐fused coat protein subunits into the same mosaic phages. To better understand the mosaic architecture, we quantified the number of peptides displayed per phage particle under genome‐encoded and plasmid‐driven expression conditions using a FlAsH‐based fluorescence strategy. In this approach, TetraCys peptide, introduced on the phage capsid either through the phage genome‐encoded display system or via plasmid‐driven expression, was specifically labeled with the biarsenical dye FlAsH‐EDT_2_ and quantified against a calibration curve generated with a synthetic CCPGCC peptide standard. This enabled estimation of the average number of TetraCys‐containing gp10 fusion proteins per phage particle, yielding 186 ± 61 and 243 ± 24 fusion proteins for genome‐ and plasmid‐driven expression, respectively, corresponding to an approximately 1.3‐fold higher incorporation for the plasmid‐driven fusion (Figure S2). The observed difference indicates that the incorporation of the two gp10 variants may be influenced by the expression levels of plasmid‐ and genome‐encoded gp10. Importantly, this balance could be further tuned using T7Select vector backbones supporting different genome‐encoded gp10 display levels. We next evaluated the functional binding of the mosaic phage using PPC1 cells, which overexpress the RPAR target receptor NRP‐1, and M21 cells, which express minimal NRP‐1 [35, 37, 38]. As T7 phage does not infect mammalian cells, PPC1 and M21 served exclusively as models for assessing NRP‐1‐dependent phage binding. Cells were incubated at 4°C with monospecific WT, RPAR, or mosaic WT/RPAR phages, followed by washing and titration of bound phage particles. As expected, WT phage exhibited negligible binding to either cell line, whereas both RPAR and WT/RPAR phages displayed significantly higher binding to PPC1 than to M21 cells (Figure 1c). Interestingly, we observed that the high valency RPAR single‐display phage exhibited increased binding to NRP1‐negative M21 cells compared with the mosaic phage. This suggests that high density of positively charged homing peptide can compromise specificity and highlights the existence of a “sweet spot” for ligand valency that balances strong target engagement with minimal background binding. Notably, while the RPAR single‐display phage showed moderately higher binding than the mosaic across both cell lines, consistent with its greater number of surface‐displayed peptides, the WT/RPAR mosaic phage maintained robust ligand‐mediated binding and high receptor specificity. These results demonstrate that co‐display under dual‐display conditions preserves functional activity of the homing peptide while potentially mitigating nonspecific interactions.

**FIGURE 1:**
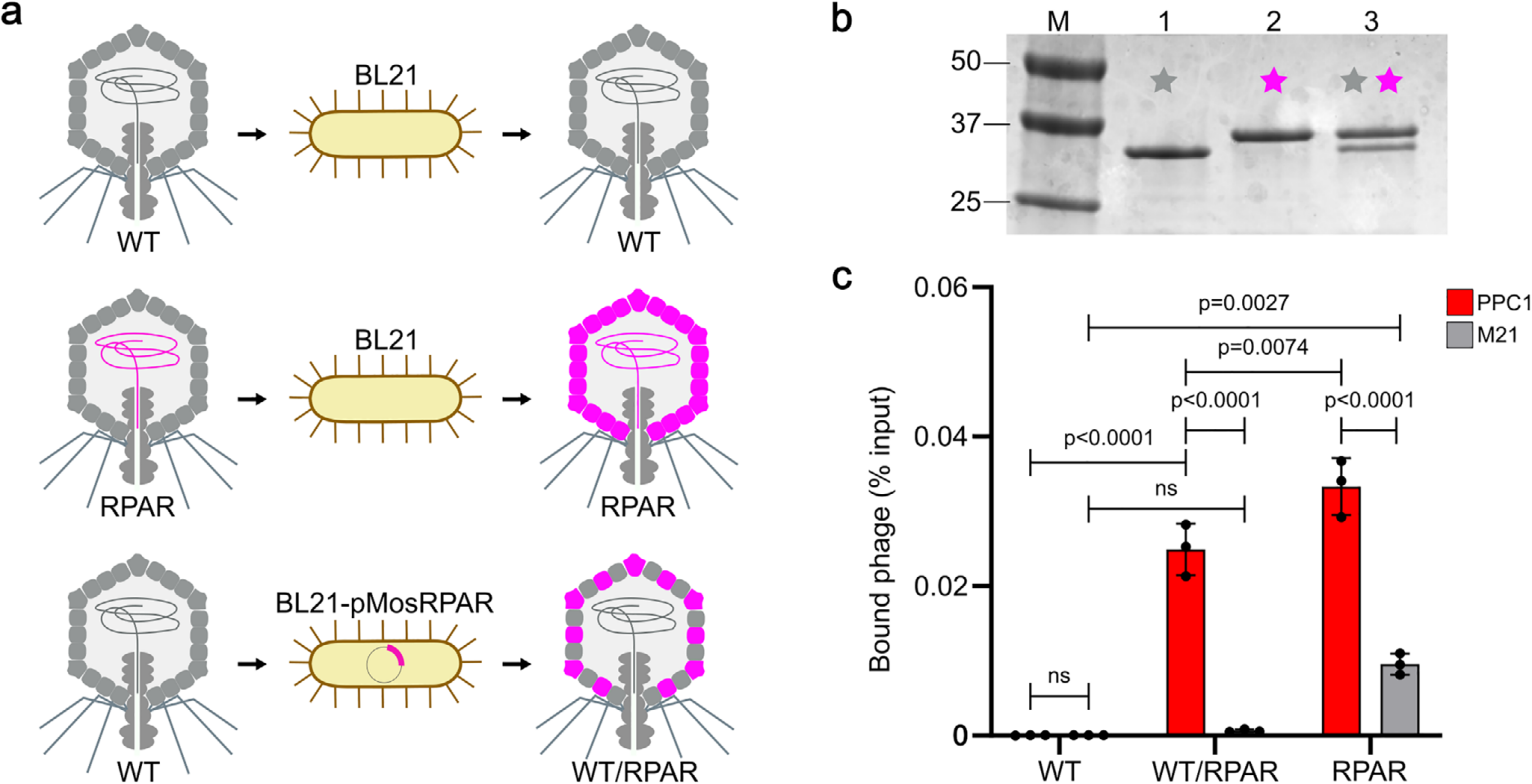
Construction, characterization, and in vitro cell binding of WT/RPAR mosaic phage. (a) Schematic of the strategy used to generate the WT/RPAR mosaic phage, combining genome‐encoded wild‐type gp10B with plasmid‐driven RPAR‐fused gp10A for dual display. (b) SDS‐PAGE analysis of CsCl‐purified T7 phage preparations stained with Coomassie Blue. Lanes: M, Precision Plus Protein Dual Color Prestained Protein Standard (Bio‐Rad); (1) WT phage; (2) RPAR phage; (3) WT/RPAR mosaic phage. Molecular weights of marker bands are indicated. (c) Phage binding is expressed as recovered titers after incubation and washing. Data represent mean ± SD from three independent experiments (*N* = 3). Statistical analysis was performed using two‐way ANOVA with Sidak's multiple comparisons test; *p* values are indicated in the figure.

### 2.3 Mosaic Phages Integrate Epitope Tags for Visualization and Tracking

Visualization and tracking of ligand‐displaying phages in a cellular context are essential for validating targeting specificity, studying receptor engagement, and integrating phage‐based tools with downstream functional assays. The HA epitope tag is widely used for antibody‐based detection in both imaging and biochemical applications. To demonstrate that mosaic phages can combine receptor‐targeting ligands with detectable tags without compromising functionality, we generated HA‐tagged single‐display and dual‐display phages, including WT/HA‐tag and RPAR/HA‐tag variants (Figure 2a). Dual‐color Western blot using anti‐T7‐tag (green) and anti‐HA‐tag (red) antibodies revealed the expected pattern of bands. WT phages showed a single green band, while HA‐tag phages displayed one band positive for both antibodies (orange), reflecting the presence of N‐terminal T7 epitope and the C‐terminal HA‐tag on the same capsid protein. In contrast, WT/HA‐tag mosaic phages exhibited two anti‐T7 antibody reactive bands, of which only one was recognized by the anti‐HA‐tag antibody. A similar pattern was observed for RPAR and RPAR/HA‐tag mosaic phages, suggesting co‐assembly of different types of coat proteins (Figure 2b). To evaluate the functional consequences of dual display in a cellular context, we incubated PPC1 and M21 cells with the mosaic and control phages, followed by confocal microscopy. PPC1 cells incubated with RPAR/HA‐tag mosaic phages showed robust HA‐tag staining at the cell surface, consistent with specific RPAR‐mediated receptor targeting, whereas M21 cells exhibited minimal signal (Figure 2c). Quantification of the mean fluorescence intensity revealed a ∼5‐fold higher signal in PPC1 cells compared to M21 cells, indicating a statistically significant difference in phage binding between the two cell lines (Figure S3). These observations indicate that mosaic phages can integrate receptor‐targeting ligands with epitope tags without compromising binding specificity to allow direct visualization and tracking in cells.

**FIGURE 2:**
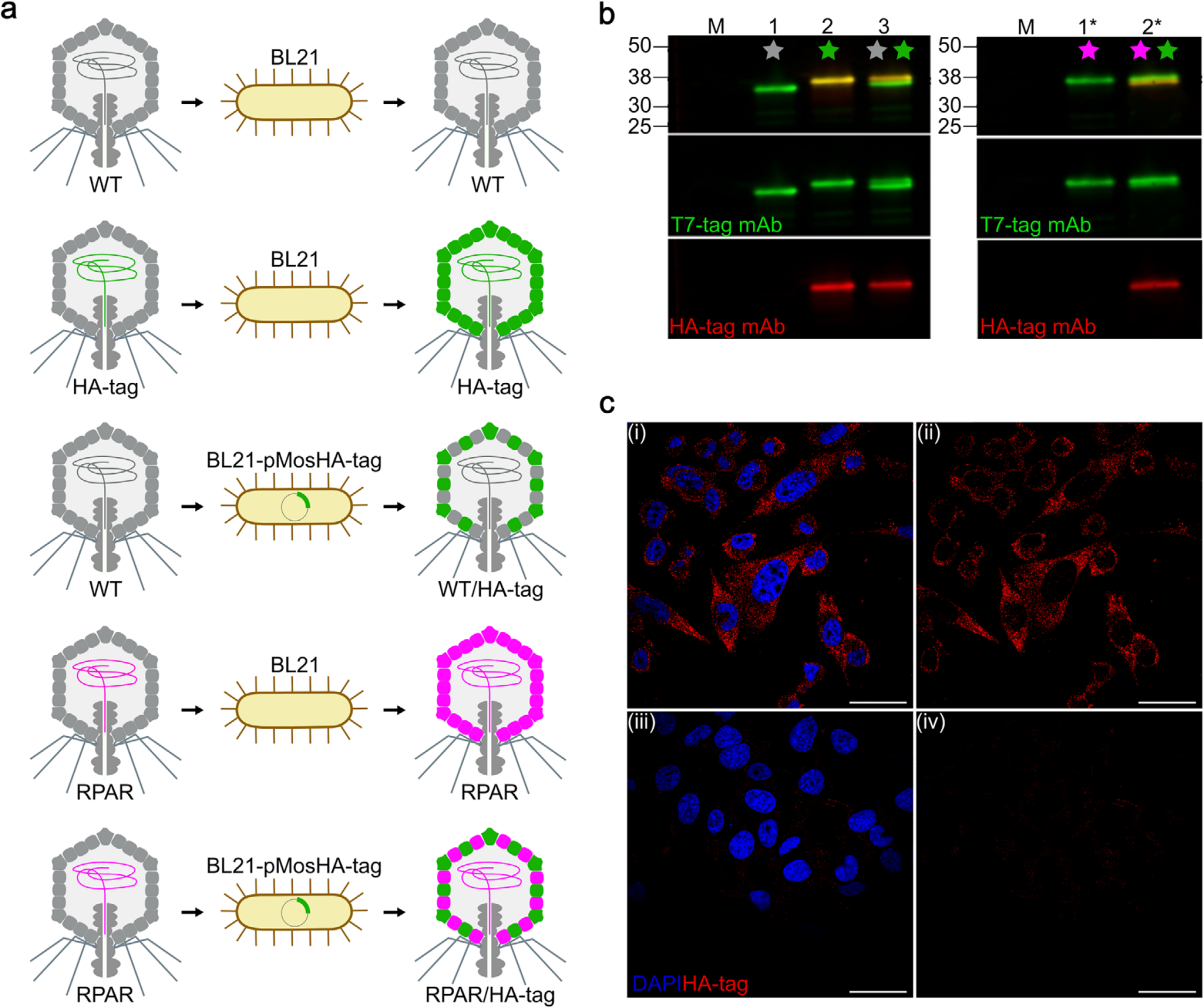
Generation and validation of HA‐tagged mosaic phage. (a) Schematic illustration of the strategy used to generate the HA‐tag mosaic phages, combining genome‐encoded wild‐type or RPAR coat proteins with plasmid‐driven HA‐tagged gp10A variants. (b) Western blot analysis of CsCl‐purified T7 phage preparations double‐stained with mouse anti‐T7‐tag mAb (green) and rabbit anti‐HA‐tag mAb (red). M: Chameleon Duo Prestained Protein Ladder (LI‐COR); (1) WT phage; (2) HA‐tag phage; (3) WT/HA‐tag mosaic phage; (1*) RPAR phage; (2*) RPAR/HA‐tag mosaic phage. Molecular weights of marker bands are indicated. (**c**) Confocal microscopy images of PPC1 cells (i, ii) and M21 (iii, iv) incubated at 37°C for 30 min with RPAR/HA‐tag mosaic phage and stained with rabbit anti‐HA‐tag mAb and secondary anti‐rabbit Alexa Fluor 546‐conjugated antibody. Blue = DAPI (nuclei); red = RPAR/HA‐tag phage. Scale bar = 20 µm.

### 2.4 Mosaic Phages Couple Cellular Targeting With Affinity Purification of Peptide Receptors

Identifying the cellular receptors of homing peptides is a major challenge using conventional approaches. Techniques such as affinity chromatography, proximity ligation proteomics, or pull‐downs often require large amounts peptide ligands, expensive reagents, and complex workflows, which can limit throughput and performance [39]. Mosaic phage particles offer a potentially transformative alternative: by co‐displaying a receptor‐targeting peptide together with an affinity tag, they can simultaneously engage target cells and provide a built‐in handle for downstream purification, thereby bypassing some of the current limitations.

Here, we first tested the ability of the well‐characterized Tstrep/Strep‐Tactin system, in which the Tstrep provides high‐affinity binding to Strep‐Tactin XT matrices, to enable selective capture of phage particles. We generated WT/Tstrep, RPAR/WT, and RPAR/Tstrep dual‐display mosaic T7 phages and first confirmed their composition by SDS–PAGE (Figure 3a,b). All samples showed two distinct coat protein bands corresponding to the wild‐type and either Tstrep‐ or RPAR‐modified subunits, confirming successful co‐assembly of both variants. In a cell‐binding assay, only RPAR‐displaying phages efficiently bound to PPC1 cells, whereas the WT/Tstrep control phages exhibited minimal interaction (Figure 3c, left). Phages recovered from the cellular lysates from the cell‐binding assay were subsequently incubated with Strep‐Tactin XT magnetic beads. Binding was observed exclusively for phages displaying the Tstrep tag, confirming selective capture via the affinity tag (Figure 3c right).

**FIGURE 3:**
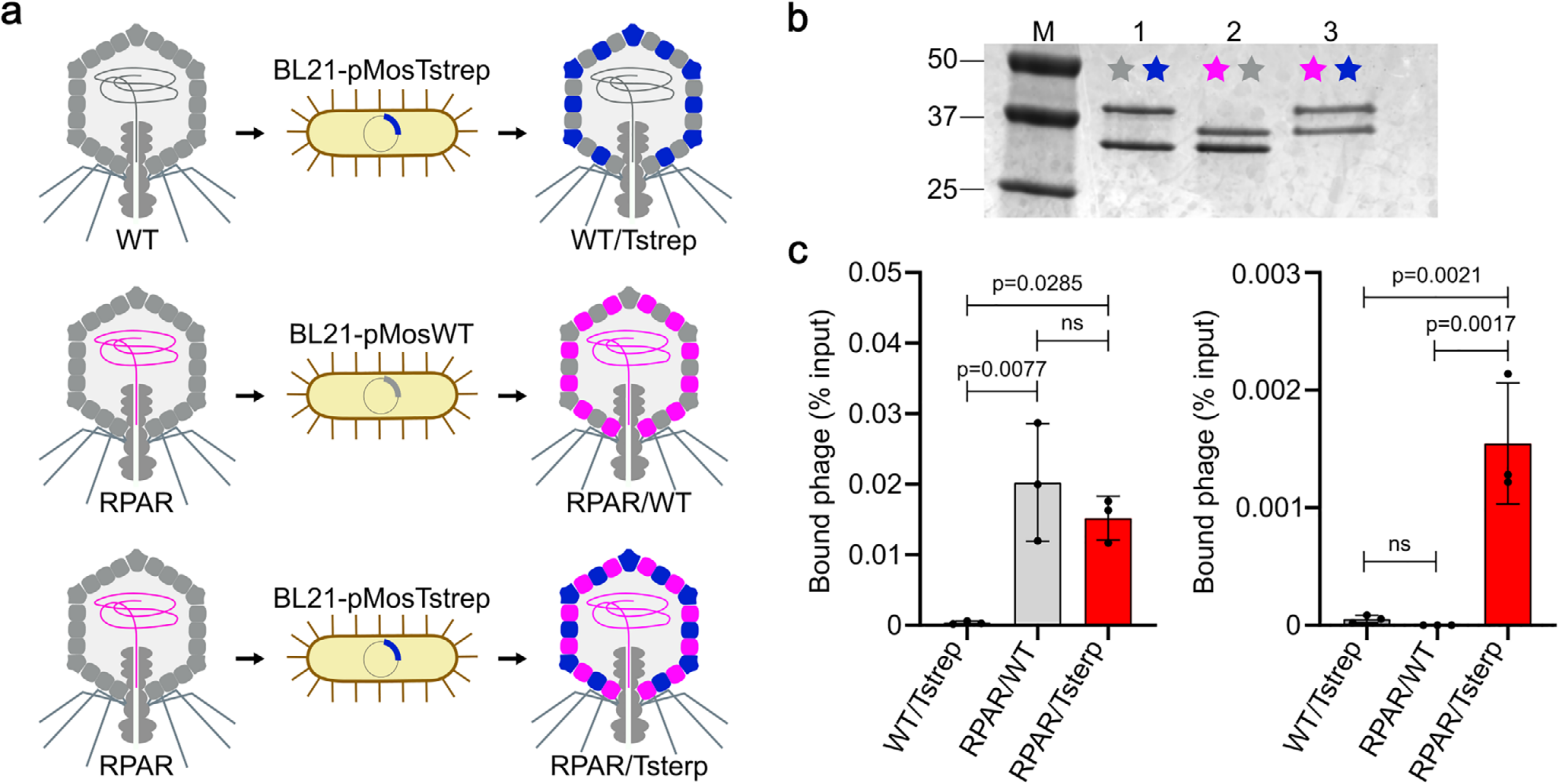
RPAR/Tstrep mosaic phages target NRP‐1 positive cells and can be pulled down using Strep‐Tactin XT beads. (a) Schematic overview of the strategy used to generate Tstrep‐displaying mosaic phages (b) SDS‐PAGE analysis of CsCl‐purified T7 phage preparations stained with Coomassie Blue. M: Precision Plus Protein Dual Color Prestained Protein Standard (Bio‐Rad); (1) WT/Tstrep mosaic phage; (2) RPAR/WT mosaic phage; (3) RPAR/Tstrep mosaic phage. Molecular weights of marker bands are indicated. (c) In vitro phage binding assay on adherent PPC1 cells and subsequent recovery of phages using Strep‐Tactin XT magnetic beads from the cellular lysates following in vitro binding assay. Data are presented as the mean ± SD from *N* = 3 independent experiments. Statistical analysis was performed using ordinary one‐way ANOVA with Tukey's multiple comparison test; *p* values are indicated in the figure.

To assess whether dual‐display mosaic phages could be used for ligand‐directed protein capture, we used Tstrep‐containing mosaic phages incorporating either the targeting RPAR peptide or non‐targeting WT and cG7c controls (Figure 4a). SDS‐PAGE analysis confirmed the expected coat protein composition, with expected size shifts observed between WT/Tstrep, cG7c/Tstrep, and RPAR/Tstrep variants (Figure 4b). We then evaluated whether RPAR/Tstrep mosaic phages could selectively capture NRP‐1 directly from PPC1 cell lysates. Strep‐Tactin XT beads coated with immobilized Tstrep‐containing mosaic phages were incubated with cellular lysates, followed by washes to remove unbound material. Bound proteins were then competitively eluted using biotin, which displaces the Tstrep tag from Strep‐Tactin XT and releases the captured proteins (Figure 4c). Western blot analysis with anti‐NRP‐1 antibody revealed a strong 130 kDa band exclusively in samples enriched with RPAR/Tstrep mosaic phages, while controls lacking the RPAR ligand showed no detectable signal (Figure 4d). Proteomics analysis confirmed NRP‐1 as the most abundantly enriched protein in RPAR/Tstrep eluates compared with cG7c/Tstrep control (Figure 4e). These results demonstrate that dual‐display mosaic phages provide a modular platform for ligand‐directed protein pull‐downs from complex biological mixtures, enabling efficient receptor identification and interactome mapping.

**FIGURE 4:**
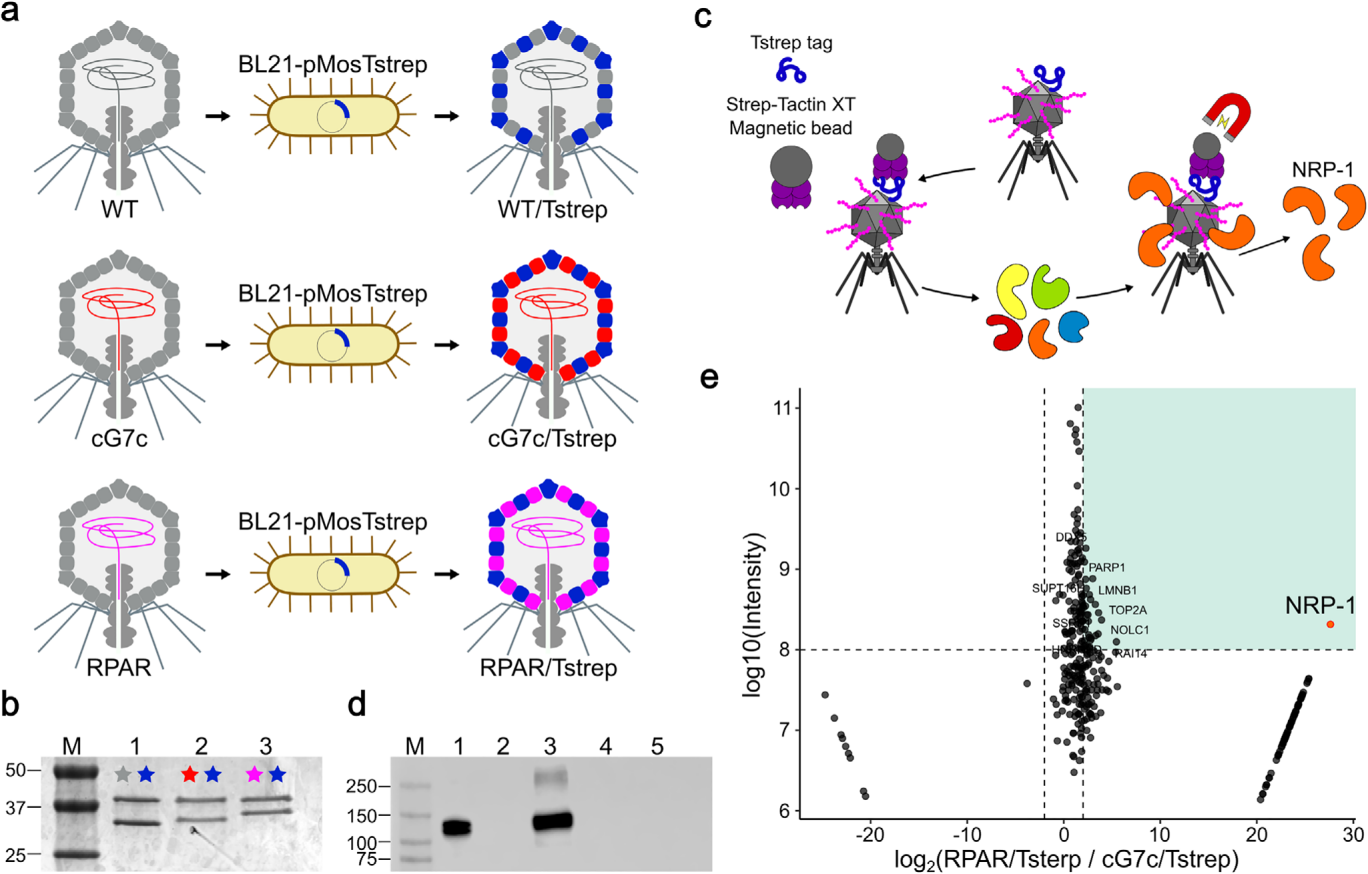
Neuropilin‐1 pull‐down using RPAR/Tstrep mosaic phage. (a) Schematic representation of the strategy used to generate Tstrep mosaic phages. (b) SDS‐PAGE analysis of CsCl‐purified T7 phage preparations stained with Coomassie Blue. M: Precision Plus Protein Dual Color Prestained Protein Standard (Bio‐Rad); (1) WT/Tstrep mosaic phage; (2) cG7c/Tstrep mosaic phage; (3) RPAR/Tstrep mosaic phage. Molecular weights of marker bands are indicated. (c) Workflow of the NRP‐1 pull‐down assay using mosaic phages and Strep‐Tactin XT magnetic beads. (d) Western blot of eluted samples probed with rabbit anti‐NRP‐1 mAb. M: Precision Plus Protein Dual Color Prestained Protein Standard (Bio‐Rad); (1) 1:20 dilution of whole protein extract used for pull‐down; (2) RPAR/Tstrep mosaic phage without protein incubation (3) RPAR/Tstrep mosaic phage incubated with protein extract; (4) cG7c/Tstrep mosaic phage incubated with protein extract; (5) WT/Tstrep mosaic phage incubated with protein extract. (e) Volcano plot comparing the proteomic profiles between RPAR/Tstrep and cG7c/Tstrep eluted samples after pull‐down assay.

### 2.5 Mosaic Phages Direct Tissue‐Specific Localization of Combinatorial Phage Libraries

To explore whether mosaic phage engineering can be used to reprogram in vivo selection, we generated a CX7C/RPAR mosaic library by combining a naïve CX7C peptide repertoire with a constant lung‐homing RPAR module (Figure 5a). Following systemic administration, phages were allowed to circulate for 30 min before extensive vascular perfusion and tissue collection. Given the rapid blood clearance reported for T7 phage [40, 41], these experimental conditions were expected to minimize the contribution of circulating phage to the recovered tissue titers. The CX7C/RPAR library showed significantly increased lung accumulation compared with the CX7C/WT control (Figure 5b). These results demonstrate that incorporation of a fixed tissue‐homing peptide is sufficient to bias the in vivo localization of a highly diverse phage library.

**FIGURE 5:**
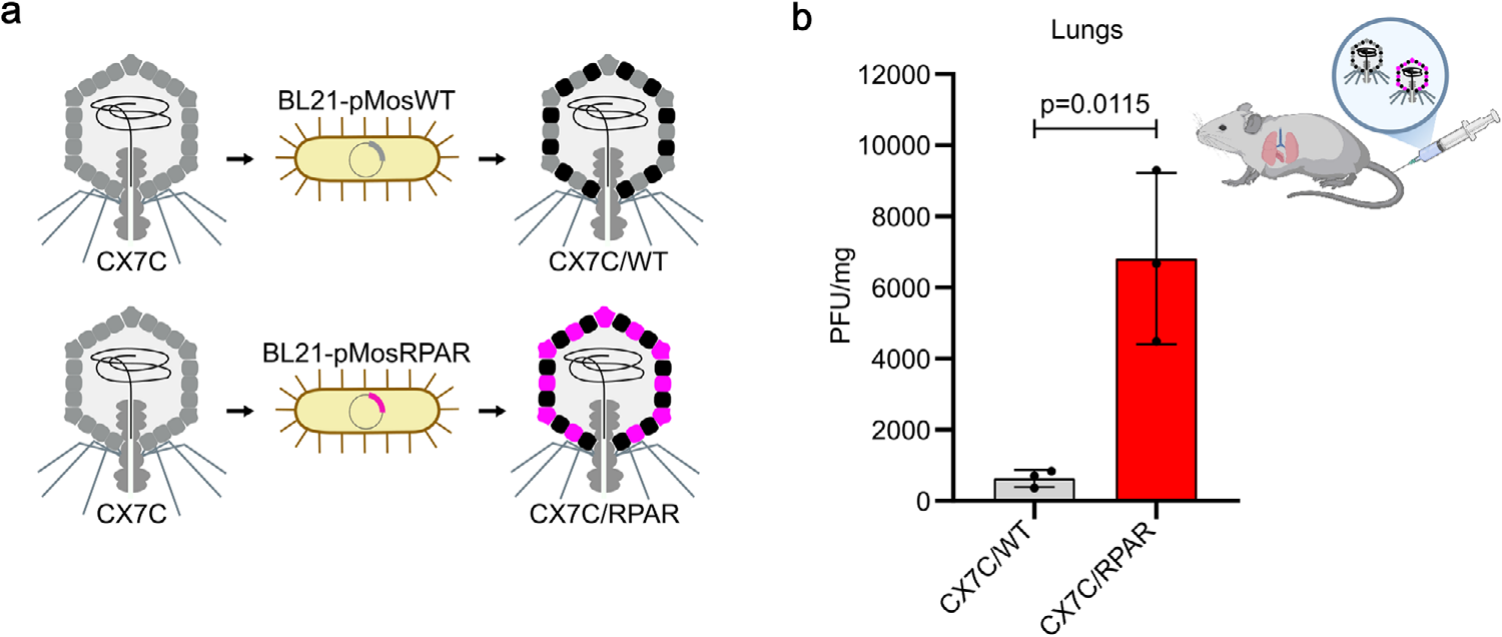
In vivo lung targeting of the CX7C/RPAR mosaic phage library. (a) Schematic illustration of the strategy used to generate CX7C/WT and CX7C/RPAR mosaic phages, combining genome‐encoded CX7C coat proteins with plasmid‐driven WT or RPAR gp10A variants. (b) Quantitative analysis of phage titers recovered from lung tissue following systemic administration in mice. Data represent mean ± SD from three animals per group (*n* = 3). Statistical analysis was performed using an unpaired two‐tailed *t*‐test; *p* value is indicated in the Figure.

## 3 Discussion

This work establishes a dual‐display mosaic T7 phage platform that converts bacteriophage T7 into a programmable system for multifunctional applications. The platform is based on the combined use of genome‐encoded and plasmid‐driven expression of peptide‐fused coat proteins, enabling simultaneous presentation of two distinct peptides on a single viral capsid. This architecture maintains phage infectivity and assembly efficiency while preserving the genetic stability of the T7 scaffold.

Functionally, mosaic phages support receptor‐specific binding, live‐cell immunodetection, and affinity‐based recovery of molecular complexes, demonstrating that orthogonal peptide functionalities can be integrated into a single phage particle without loss usability. Together, these features establish a modular T7‐based system in which targeting and biochemical handle functions can be independently programmed at the level of capsid assembly.

A key application of this approach is in addressing a long‐standing challenge in peptide ligand discovery: the identification of receptors for peptides selected by phage display. Although phage selection routinely yields affinity ligands for cells, tissues, or purified targets, identification of their molecular binding partners remains technically demanding [42, 43]. Established approaches, including affinity pull‐down, proximity labeling, and mass spectrometry‐based proteomics often require extensive optimization and can be limited by nonspecific interactions, low‐abundance targets, and transient protein‐ligand complexes targets [44, 45]. The mosaic T7 design addresses these limitations by coupling two orthogonal functions within a single particle. One peptide mediates high‐avidity binding under near‐physiological conditions, while the second provides an independent biochemical handle for stringent enrichment and downstream proteomic analysis. This configuration enables integrated workflows in which target engagement and receptor capture are performed using the same molecular scaffold, thereby directly linking ligand selection to receptor identification. As a result, experimental complexity is reduced and receptor mapping becomes more tightly coupled to functional ligand discovery. Once established, the mosaic T7 architecture provides a versatile experimental platform that can be readily adapted to different applications by exchanging the displayed functional modules. The same phage scaffold can therefore support multiple research workflows, including targeted affinity capture and tissue‐directed library screening, while retaining the simplicity and scalability of bacterial phage production. This modularity is further supported by the gp10A plasmid backbone, which allows alternative ligands or functional domains to be rapidly introduced without modification of the viral genome. The flexibility of this architecture enables straightforward integration with chemoproteomic platforms, such as ligand‐based receptor capture and proximity labeling strategies, including APEX2 and TurboID, which have significantly advanced interactome mapping in other systems [46, 47, 48].

Extending the mosaic phage concept to in vivo selection, it can also be used to reprogram the spatial context of combinatorial library screening. By combining a naïve peptide repertoire with a constant homing module, the library is no longer distributed uniformly across the organism but is instead biased toward a predefined anatomical or physiologically‐defined compartment. This restriction effectively relocates the search space, enabling secondary selection events to occur within a tissue‐specific microenvironment while preserving global combinatorial diversity. In this configuration, tissue homing and molecular recognition become separable design layers, allowing in vivo phage display to be directed toward specific biological niches rather than the whole organism. This strategy is particularly relevant for complex tissues and pathological states characterized by post‐target heterogeneity. In the case of tissue‐penetrating peptides such as iRGD and related CendR‐class systems [49], it may enable interrogation of molecular diversity beyond vascular entry barriers, extending ligand discovery into previously inaccessible post‐barrier compartments.

Although this work establishes the feasibility of the mosaic T7 platform, several design considerations should be noted. As with conventional phage display systems, successful peptide incorporation depends on intrinsic sequence properties, including length, charge, and amino acid composition. In the mosaic format, an additional parameter emerges: compatibility between co‐displayed peptides, which may influence capsid assembly, ligand accessibility, and overall biological performance. This parameter may become particularly relevant when the mosaic strategy is applied to highly diverse libraries, where differential compatibility with the constant plasmid‐derived module could influence clone representation during amplification. A further consideration is the trans‐complementation strategy used for the plasmid‐derived module during phage amplification. While this approach enables rapid exchange of functional elements without re‐engineering the phage genome, it decouples the additional functionality from the phage genotype, limiting direct genetic tracking of the secondary module during downstream selection cycles. Despite these constraints, the modular architecture offers substantial flexibility for future development. Beyond receptor mapping and affinity purification, the same platform can be extended to incorporate catalytic domains, biosensing elements, or other functional modules, positioning mosaic T7 phages as adaptable building blocks for multifunctional nanostructures and engineered biological systems [50, 51].

## 4 Conclusion

We established a dual‐display mosaic phage platform that expands bacteriophage T7 into a programmable nanoscale system for multifunctional applications. By enabling the co‐presentation of two independent peptide functions on a single capsid, the design preserves phage activity while integrating receptor targeting with molecular detection and affinity‐based protein enrichment. Beyond enabling dual functionalization, the platform provides a generalizable framework for constructing tissue‐restricted and compositionally defined phage libraries, in which in vivo selection can be biased toward specific anatomical compartments prior to secondary peptide discovery.

More broadly, mosaic T7 phages support programmable combinations of targeting and functional readouts, enabling multiparametric selection strategies, hybrid library design, and spatially informed interactome mapping in complex biological systems.

## 5 Methods

### 5.1 Bacterial Strains

*Escherichia coli* BLT5403 (EMD Millipore), harboring the p5403 plasmid encoding the wild‐type gp10A major capsid protein, was used for plasmid extraction and engineering of the p5403‐MCS construct. The *E. coli* BLT5615 strain (EMD Millipore) was used for phage plaque titering. *E. coli* BL21 (EMD Millipore) was employed for cloning, phage amplification, and production of mosaic phages. All strains were maintained as glycerol stocks at ‐80°C and cultured in LB medium supplemented with 100 µg/mL ampicillin when carrying plasmid constructs.

### 5.2 p5403 Plasmid Extraction and Sequencing

The p5403 plasmid was purified from *E. coli* BLT5403 using the Qiagen Plasmid Maxi Kit (Qiagen) according to the manufacturer's instructions. Briefly, a single glycerol stock was inoculated in 500 mL of LB medium containing 100 µg/mL ampicillin and incubated overnight at 37°C with shaking at 200 rpm. Cells were harvested by centrifugation at 6000 × *g* for 15 min at 4°C, and plasmid DNA was extracted via alkaline lysis, column purification, and ethanol precipitation. Since no reference sequence for p5403 was available, de novo sequencing was performed using overlapping Sanger sequencing reactions. Primers 5403_Up_1_C01 (5’‐CTGTCGGACAGATAGCTACTCTGCG–3’), 5403_Up_2_D01 (5’‐GGGAAGATGGGTGCCGAGTGGG‐3’), and 5403_Down_1_D02 (5’‐TTTTTTAATCAGACGTCAGCCGTCAG‐3’) were used to generate reads, which were assembled into a 5126 bp contiguous plasmid map containing the gp10A coding region (4022–5059 bp). The C‐terminal region was selected for insertion of a multiple cloning site (MCS).

### 5.3 Engineering of p5403 Plasmid With MCS

To introduce an MCS downstream of the gp10A, 6 µg of p5403 plasmid DNA was digested with FastDigest EcoRI (Thermo Scientific) at 37°C for 30 min and heat‐inactivated at 80°C for 10 min. DNA ends were blunted with Klenow Fragment (Thermo Scientific) in the presence of 2 mM dNTPs (Thermo Scientific) at 37°C for 30 min and heat‐inactivated at 75°C for 10 min. Linearized plasmid was gel‐purified using FavorPrep GEL/PCR Kit (Favorgen). A 297 bp MCS fragment containing EcoRI, HindIII, NotI and XhoI sites was PCR‐amplified using Phusion High‐Fidelity DNA Polymerase (Thermo Scientific) with primers MCS_fwr (5’‐CGTTATGGGCTTTGAGGTTG‐3’) and MCS_rev (5’‐CGCTCGAGCCTACTTATTCACTATTAAAGCTTGCGGCCGCGGAGCTCGAATTCTTCTTCCACTTTAAAGACCACTGC‐3’). PCR conditions were (98°C for 30 s; 2 cycles: at 98°C for 10 s, 45–55°C for 10 s, 72°C for 1 min; 32 cycles: 98°C for 10 s, 65°C for 10 s, 72°C for 10 s; final extension: 72°C for 10 min). PCR products were phosphorylated using T4 Polynucleotide Kinase (Thermo Scientific) at 37°C for 30 min, followed by heat inactivation at 75°C for 10 min, digested with FastDigest KpnI (Thermo Scientific) at 37°C for 3 h, and purified. Ligation was performed overnight at room temperature using T4 DNA Ligase (Thermo Scientific) at 1:12 vector:insert molar ratio. Ligated products were transformed into NovaXG chemically competent *E. coli*, plated on LB‐ampicillin plates, and screened by colony PCR and Sanger sequencing. Clones containing the correct MCS sequence in‐frame with gp10A were selected.

### 5.4 Peptide Cloning Into p5403‐MCS and *E. coli* BL21 Strain Transformation

For peptide cloning, 1 µg of p5403‐MCS was double‐digested with FastDigest EcoRI and HindIII (Thermo Scientific) in 1× FastDigest buffer at 37°C for 1 h and heat‐inactivated at 80°C for 10 min. Linearized plasmid was dephosphorylated with FastAP Thermosensitive Alkaline Phosphatase (Thermo Scientific) at 37°C for 10 min and heat‐inactivated at 65°C for 10 min. Complementary 5’‐phosphorylated oligos (IDT; sequences in Table S1) were annealed in vitro by heating to 96°C for 5 min and cooling slowly to room temperature. Ligation reactions (1:3 vector:insert molar ratio) were performed in 20 µL final volume with T4 DNA Ligase (Thermo Scientific) and incubated overnight at room temperature. Ligation mixtures were purified using DNA Clean & Concentrator Kit (Zymo Research) and 2 µL were transformed into *E. coli* BL21 electrocompetent cells via Gene Pulser II Electroporation System (Bio‐Rad) at 2.5 kV with 0.2 cm cuvettes (Bio‐Rad). Transformed cells were recovered in LB medium for 45 min at 37°C with shaking (200 rpm) and plated on LB‐ampicillin agar. For each construct, 5–10 colonies were screened by colony PCR, and positive clones were confirmed by Sanger sequencing using primers T7_up (5’‐AGCGGACCAGATTATCGCTA‐3’) and T7_Down (5’‐AACCCCTCAAGACCCGTTTA‐3’). Verified clones were used to generate *E. coli* BL21 variants BL21‐pMosWT, BL21‐pMosRPAR, BL21‐pMosHA‐tag, and BL21‐pMosTstrep stored at ‐80°C in 20% glycerol.

### 5.5 Phage Cloning, Amplification, and Purification

Single‐peptide display phages (WT, G7, RPAR and HA‐tag) were produced using the T7Select System (EMD Millipore). Complementary 5’‐phosphorylated oligos (IDT; sequences in Table S2) were annealed and ligated into EcoRI/HindIII Vector Arms at 1:4 vector:insert molar ratio. Ligation reactions were incubated overnight at room temperature and subsequently packaged in vitro using T7 Packaging Extract according to the manufacturer's protocol (EMD Millipore). Packaged phages were diluted in LB medium, and plaque assays were performed with *E. coli* BLT5615 in top agar containing 1 mM IPTG. Plates were incubated overnight at room temperature, and individual plaques were screened by PCR and Sanger sequencing. Positive clones were amplified in *E. coli* BL21 or BL21‐p5403 cultures (200 mL LB supplemented with ampicillin or without antibiotic, respectively) infected at OD600 = 0.5 with 50 µL of phage stock. Cultures were incubated at 37°C with shaking (200 rpm) until complete lysis (1.5 h). NaCl was added to 0.5 M final concentration, and lysates were clarified by centrifugation (11,000 × *g*, 10 min, 4°C), phages were then precipitated with 10% (w/v) PEG8000, followed by centrifugation (8000 × *g*, 10 min, 4°C). Pellets were resuspended in PBS and purified by CsCl gradient ultracentrifugation (Beckman L7‐55, SW60Ti, 40,000 rpm, 45 min, 4°C). Purified phages were recovered by syringe, dialyzed against PBS using Slide‐A‐Lyzer 3.5 kDa MWCO cassettes (Thermo Scientific) with two buffer changes (1 h each, room temperature), quantified by plaque assay and stored at 4°C.

### 5.6 FlAsH‐Based Absolute Peptide Quantification

The number of peptides displayed per phage under genome‐encoded and plasmid‐based expression conditions was determined by measuring fluorescence generated by the reaction between FlAsH‐EDT2 and the TetraCys tag (CCPGCC). Stock solutions of 1,2‐ethanedithiol (EDT) (>99%, Merck) and TC‐FlAsH (Invitrogen) were prepared in DMSO at 25 mM and 2 mM, respectively. FlAsH activation was performed by incubating EDT and TC‐FlAsH at a 2:1 volume ratio for 10 min at room temperature, followed by dilution to final concentrations of 200 µM FlAsH and 5 mM EDT. Synthetic Ac‐CCPGCC (TAG Copenhagen A/S) peptide was dissolved in PBS to obtain a 2 mM stock solution. FlAsH labeling reactions were carried out under reducing conditions in a final volume of 500 µL, containing 20 µM FlAsH–EDT_2_, 20 µM peptide, and 150 mM β‐mercaptoethanol, and incubated overnight at room temperature under gentle rotation in the dark. Purified phage preparations (WT/WT, TetraCys/WT, and mosaic WT/TetraCys) were incubated with 2.5 µM FlAsH–EDT_2_ and 150 mM β‐mercaptoethanol under the same conditions, using 7.0 × 10^10^ phage PFU per reaction. Labeled phages were dialyzed against PBS using 3.5 kDa MWCO Slide‐A‐Lyzer cassettes (Thermo Scientific) to remove unbound dye. A calibration curve was generated from the FlAsH‐labeled synthetic Ac‐CCPGCC peptide. Serial dilutions of the peptide reaction and identically processed PBS controls were used for background subtraction. Fluorescence was measured in 96‐well plates (100 µL per well) using an EnSight Multimode Plate Reader (PerkinElmer) with 508 nm excitation and 528 nm emission and analyzed using GraphPad Prism to determine the number of peptide molecules per phage.

### 5.7 SDS‐PAGE and Western Blot

Phage protein concentrations were determined using a spectrophotometer with Bradford assay. For SDS‐PAGE, 1 µg of phage protein was denatured in Laemmli buffer with 10% β‐mercaptoethanol, heated at 95°C for 5 min, and loaded onto 4–20% Mini‐PROTEAN TGX gels (Bio‐Rad). Gels were run at 80 V for 10 min followed by 110 V for 1 h. For Coomassie staining, gels were incubated overnight in a 0.5% (w/v) Coomassie Brilliant Blue G‐250 (Merck) solution prepared in 40% (v/v) methanol and 10% (v/v) acetic acid in Milli‐Q water. Gels were subsequently destained in a solution containing 25% (v/v) methanol and 10% (v/v) acetic acid in Milli‐Q water and imaged on an Odyssey M system (LI‐COR). For dual‐color western blot, proteins were transferred to membranes (Immobilon‐FL transfer membrane PVDF 0.45 µm, Merck Millipore) using the Trans‐Blot Turbo system (Bio‐Rad). Membranes were blocked in Intercept TBS Blocking Buffer (LI‐COR) for 1 h at room temperature, then incubated overnight at 4°C with mouse anti‐T7‐tag (1:10,000; Novagen) and rabbit anti‐HA‐tag (1:1000; Cell Signaling Technology) antibodies. After washes in TBS + 0.1% Tween‐20 (TBS‐T), membranes were incubated for 1 h at room temperature with IRDye 800CW anti‐mouse (1:10,000) and IRDye 680LT anti‐rabbit (1:40,000) secondary antibodies (LI‐COR), washed and imaged on an Odyssey CLx system (LI‐COR). For Neuropilin‐1 detection, eluates from pull‐down assay were separated by SDS‐PAGE, transferred to PVDF membranes, blocked in 5% low‐fat milk/TBS‐T, incubated overnight with monoclonal rabbit anti‐Neuropilin‐1 (1:1000; Abcam) in 3% low‐fat milk/TBS‐T, washed and probed with HRP‐conjugated anti‐rabbit secondary antibody (1:10,000; BioLegend). Chemiluminescent signals were visualized using SuperSignal West Pico PLUS substrate (Thermo Scientific) with Odyssey XF system (LI‐COR).

### 5.8 Cell‐Binding Assay and Immunofluorescence

PPC1 (human prostate carcinoma) and M21 (human melanoma) cells were cultured in DMEM (4.5 g/L glucose, no L‐glutamine; Lonza) with 10% FBS, 100 U/mL penicillin, and 100 µg/mL streptomycin at 37°C in 5% CO_2_. For binding assay, 70,000 cells/well were seeded in 24‐well plates, grown overnight, washed with cold DMEM + 1% BSA, and incubated with 5 × 10^7^ PFU of phages in 1 mL at 4°C for 1 h with gentle rocking. After six washes with 1 mL DMEM + 1% BSA, cells were lysed in 1 mL lysis buffer (LB + 1% NP‐40) and phage titers were determined by plaque assay. Binding efficiency was calculated as: recovered phage PFU/input phage PFU. For immunofluorescence, 50,000 cells/well were seeded on glass coverslips, incubated with 5 × 10^7^ PFU of phages at 37°C for 30 min, washed with DMEM, fixed in 4% PFA for 10 min, blocked in 5% goat serum + 0.2% Triton X‐100 for 45 min, and stained with rabbit anti‐HA‐tag antibody (1:200; Cell Signaling Technology) for 1 h, followed by Alexa Fluor 546 goat anti‐rabbit (1:500; Thermo Scientific) and DAPI. Coverslips were mounted with Fluoromount‐G, and images were acquired with an Olympus FV1200MPE (Evident Scientific) confocal microscope using FV10‐ASW 4.0 software and analyzed with Imaris ×64 version 8.2.1 (Oxford Instruments).

### 5.9 Cell‐binding Assay and Magnetic Beads Affinity Capture of Mosaic Phages

The in vitro cell‐binding assay was performed as described above using PPC1 cells. After cell lysis and phage recovery, 900 µL of the recovered phage suspension was incubated for 2 h at 4°C with gentle rotation with 20 µL of MagStrep Strep‐Tactin XT beads (IBA Lifesciences) prewashed in PBS. Beads were then washed six times with 1 mL PBS + 0.1% BSA to remove unbound particles. Bound phages were eluted in 400 µL of 1 × BXT Elution Buffer (IBA Lifesciences) in PBS + 0.1% BSA, and eluates were quantified by plaque assay.

### 5.10 Cell Lysate Preparation and Neuropilin‐1 Pull‐Down Using Mosaic Phage

PPC1 cells (80%–90% confluent) were washed twice in PBS, scraped, pelleted at 230 × g for 6 min, resuspended in ice‐cold PBS, pelleted again, and lysed in 100 µL modified RIPA buffer (50 mM Tris pH 7.4, 5 mM EDTA, 250 mM NaCl, 0.1% Triton X‐100) supplemented with protease and phosphatase inhibitors (Thermo Scientific). Lysates were incubated on ice for 30 min, centrifuged at 14,000 × *g* for 10 min at 4°C, and supernatants were collected and quantified by Bradford assay. For pull‐down assay, 30 µL MagStrep Strep‐Tactin XT beads (IBA Lifesciences) were washed three times with 1 mL PBS, incubated with 5 × 10^7^ PFU of phages in 1 mL PBS + 0.1% NP‐40 for 2 h at 4°C with rotation, washed six times with 1 mL PBS + 0.1% NP‐40. Beads were then incubated with 450 µL protein extract (4 µg/µL) for 1 h at 4°C with rotation, followed by six washes with 1 mL PBS + 0.1% NP‐40. Bound phage‐protein complexes were eluted in 30 µL with 1X BXT Elution Buffer (IBA Lifesciences) in PBS + 0.1% NP‐40. The eluate was used for subsequent Western blot and proteomic analyses.

### 5.11 Liquid Chromatography and Tandem Mass‐Spectrometry Analysis (LC‐MS/MS)

Proteins were precipitated with 20% TCA and 0.8 mg/mL sodium deoxycholate, collected by centrifugation, and washed subsequently with acetone and methanol. Dried protein pellets were resuspended in 7 M urea, 2 M thiourea, 100 mM ammonium bicarbonate, and 20 mM methylamine buffer. Dissolved proteins were reduced with 10 mM DTT for 30 min and alkylated with 30 mM chloroacetamide for 30 min in the dark, before adding proteomics‐grade Lys‐C protease (1:100 ratio over substrate; Fujifilm Wako Pure Chemical) for pre‐digestion at 25°C for 2 h. Pre‐digested samples were diluted 1:5 with 100 mM ammonium bicarbonate before adding dimethylated porcine trypsin (1:50 ratio over substrate; Sigma Aldrich) for 16–18 h proteolysis at 25°C. The resulting tryptic peptide samples were acidified with 1% trifluoroacetic acid before desalting using in‐house prepared C18 (3M) solid phase extraction tips. The Dionex Ultimate 3000 RSLCnano system was used as a front‐end reverse chromatography system for the peptide separation before the MS analysis. Prepared tryptic peptide samples were loaded onto a trap‐column (0.3 × 5 mm; 5 µm C18 particles, Dionex), then injected into an in‐house packed (3 µm C18; Dr Maisch) analytical emitter‐column (50 cm × 75 µm; MS Wil, CoAnn Technologies). Peptides were separated with 60 min 8–42% buffer B gradient (buffer A: 0.1% formic acid; buffer B: 80% acetonitrile and 0.1% formic acid) and delivered directly into a Q Exactive HF (Thermo Scientific) mass spectrometer via a nano‐electrospray source, using positive mode and spray voltage of 2.5 kV. Each 350–1400 m/z full MS scan by the mass spectrometer was followed by higher‐energy collisional dissociation fragmentation (normalized collision energy set at 26) and MS/MS analysis of the 12 most intense ions. The following settings were used for MS and MS/MS analysis, respectively: resolution *R* = 60,000 and 30,000, the ion number target values 3,000,000 and 100,000, and the injection times 50 and 41 ms. Ion charge was set to *z* = +2 to +5, MS/MS isolation window to 1.6 m/z, and dynamic exclusion to 20 s. Raw MS files were processed with the MaxQuant software package (version 2.2.0.0) [52], using default search parameters. Methionine oxidation and protein N‐terminal acetylation were set as variable modifications, and cysteine carbamidomethylation as a fixed modification. Human and T7 bacteriophage proteome databases, downloaded in September 2023 and June 2025, respectively, and a common contaminating proteins database [53], were used as target databases in the software search.

### 5.12 In Vivo Administration and Tissue Recovery of Mosaic Phages

The animal experiments were performed with healthy mice, according to animal experimentation protocols approved by Estonian Ministry of Agriculture, Committee of Animal Experimentation (permits #159 and #299). CX7C naïve phage library generated in our laboratory was combined with plasmid‐driven expression of the RPAR peptide, yielding CX7C/RPAR and CX7C/WT libraries. Phages were purified and quantified as described above. Subsequently, 100 µL of phage suspension (5 × 10^9^ total PFU; stock concentration 5.0 × 10^10^ PFU/mL) were administered via tail vein injection to 7‐week‐old female BALB/c mice (*n* = 3 mice per experimental group). Phages were allowed to circulate for 30 min, after which mice were anesthetized by intraperitoneal injection of ketamine (90 mg/kg)/dexmedetomidine (0.25 mg/kg) prepared in 0.9% saline, followed by intracardial perfusion with 20 mL of PBS. Organs were then washed with PBS, weighed, and collected on ice in snap‐cap tubes containing 1 mL of LB supplemented with 1% NP‐40. Tissues were homogenized using a handheld tissue homogenizer (Omni Tissue Homogenizer TH, Omni‐Inc) equipped with disposable 7 mm hard‐tissue probes (Omni Tip, Omni‐Inc). Recovered phages were quantified by plaque assay and expressed as plaque‐forming units per mg of tissue (PFU/mg).

### 5.13 Statistical Analysis

Statistical analysis was performed using GraphPad Prism software (version 8.0.2). Data are presented as mean ± SD. Comparisons between two groups were performed using an unpaired two‐tailed Student's t‐test. For comparisons involving more than two groups, one‐way ANOVA was used. Two‐way ANOVA was applied for experiments involving two independent variables. A *p* value of < 0.05 was considered statistically significant.

## Author Contributions

**Luca Marchetti**: conceptualization, writing – original draft, investigation, methodology, validation, visualization, writing – review and editing, formal analysis, data curation. **Kristina Põšnograjeva**: writing – review and editing, visualization, validation, methodology, investigation, formal analysis. **Prakash Lingasamy**: conceptualization, investigation, writing – review and editing, methodology, validation, visualization, formal analysis, data curation. **Karlis Pleiko**: writing – review and editing, visualization, validation, methodology, data curation, formal analysis**. Ivar Ilves**: formal analysis. **Tambet Teesalu**: conceptualization, funding acquisition, writing – review and editing, project administration, supervision, investigation, resources, writing – original draft.

## Conflicts of Interest

Tambet Teesalu is the inventor of patents on iRGD and CendR peptides.

## Supporting information




**Supporting File**: biot70322‐sup‐0001‐SuppMat.docx.

## Data Availability

The mass spectrometry proteomics data have been deposited to the ProteomeXchange Consortium via the PRIDE partner repository with the dataset identifier PXD080235 (https://doi.org/10.6019/PXD080235).
