# Supplementary material for: Engineering Mosaic T7 Bacteriophages for Dual Peptide Display to Enable Cellular Targeting and Ligand‐Directed Protein Capture
Source: Biotechnol J. 2026 Oct 28;21(11):e70322. doi: 10.1002/biot.70322 (PMC13717207; doi:10.1002/biot.70322)
Supplement: Supplementary file 1 [file BIOT-21-e70322-s001.docx]

**Mosaic T7 phage platform for peptide co-display, cellular targeting, and ligand-directed protein capture**

Luca Marchetti^1^, Kristina Põšnograjeva^1^, Prakash Lingasamy^1^, Karlis Pleiko^1,3^, Ivar Ilves^2^, Tambet Teesalu^1,4,#^

^1^ Laboratory of Precision and Nanomedicine, Institute of Biomedicine and Translational Medicine, University of Tartu, Ravila 14b, 50090 Tartu, Estonia.

^2^ Institute of Technology, University of Tartu, Nooruse tn 1, 50411 Tartu, Estonia

^3^ Latvian Institute of Organic Synthesis, Aizkraukles 21, Riga, LV1006 Latvia

^4^ Materials Research Laboratory, University of California, Santa Barbara, CA 93106, USA.

^#^Corresponding author: Tambet Teesalu, tambet.teesalu@ut.ee

.

**Supplementary Material**

**Figure S1. Schematic maps of mosaic plasmid constructs.**

Vector maps generated with Vector NTI showing p5403, the modified p5403-MCS containing a multiple cloning site (MCS) at the C-terminus of the gp10 capsid gene, and the derived constructs pMosWT, pMosRPAR, pMosHA-tag, pMosTstrep, pMosTetraCys. Each map indicates the T7 promoter, gp10A coding region, plasmid origin of replication (ORI), the β-lactamase resistance gene (AmpR), and the EcoRI and HindIII restriction site used for cloning. Amino acid sequences of genome-encoded gp10B and plasmid-driven gp10A coat proteins are shown. The two sequences are largely identical, differing only at the C-terminal region. The N-terminal region (highlighted in green) corresponds to the T7-tag epitope recognized by the anti–T7-tag antibody, while the C-terminal residues corresponding to the EcoRI cloning site are underlined.

**Figure S2. FlAsH-based quantification of peptide valency on mosaic phages**.

(**a**) Schematic illustration of the strategy used to generate TetraCys mosaic phages, combining genome-encoded wild-type or TetraCys coat proteins with plasmid-driven TetraCys gp10A variants. (**b**) SDS–PAGE analysis of CsCl-purified T7 phage preparations. Left: fluorescence imaging of FlAsH-labeled phages; right: Coomassie Blue staining of the same samples. Lanes: M, Precision Plus Protein™ Dual Color Prestained Protein Standard (Bio-Rad); (1) WT/WT phage; (2) WT/TetraCys mosaic phage; (3) TetraCys/WT phage. Molecular weights of marker bands are indicated. (**c**) Calibration curve generated using FlAsH–EDT₂–labeled synthetic CCPGCC peptide. Fluorescence of labeled phage samples (TetraCys/WT and WT/TetraCys) were measured using a plate reader and plotted onto the calibration curve to determine the absolute number of peptides displayed per virion. Error bars represent mean ± SD from three independent experiments (N=3).

**Figure S3. Quantification of phage-associated fluorescence in PPC1 and M21 cells.**

Fluorescence intensity was quantified from confocal microscopy images of PPC1 and M21 cells incubated with RPAR/HA-tag mosaic phage and stained with anti-HA antibody and Alexa Fluor 546-conjugated secondary antibody. Image analysis was performed using Fiji ImageJ software. Data are presented as mean ± SD (n = 10 fields per experiment; three independent experiments). Statistical analysis was performed using an unpaired two-tailed t-test; p-value is indicated in the Figure.

**Table S1. Oligos used for p5403-MCS cloning**

**Table S2. Oligos used for T7 phage cloning**
